# Development of solid-state fluorescence lifetime standards for clinical applications using dyed epoxy resins

**DOI:** 10.1117/1.JBO.31.11.113503

**Published:** 2026-03-24

**Authors:** Dario Angelone, Keela Hughes, Hasti Yavari, Sara Eugenia Garduño Gómez, Brian C. Wilson, Stefan Andersson-Engels, Katarzyna Komolibus, Sanathana Konugolu Venkata Sekar

**Affiliations:** a Tyndall National Institute, Biophotonics@Tyndall, Cork, Ireland; b University College Cork, School of Physics, Cork, Ireland; c BioPixS Ltd. (Biophotonics Standards), Cork, Ireland; d University Health Network, Princess Margaret Cancer Centre, Toronto, Ontario, Canada

**Keywords:** fluorescence lifetime imaging, standardization, phantoms, calibration

## Abstract

**Significance:**

Fluorescence lifetime imaging (FLIm) offers label-free contrast based on intrinsic tissue properties, making it a promising tool for clinical diagnostics and intraoperative guidance. However, the lack of robust, reproducible standards for system validation limits cross-platform comparability, impedes quality assurance, and hinders clinical translation.

**Aim:**

We aim to develop and characterize a set of stable solid-state fluorescence lifetime (FLT) standards using dyed epoxy resins, with the goal of enabling reliable calibration, benchmarking, and validation of FLIm systems in both research and clinical environments.

**Approach:**

A series of solid standards incorporating different dyes were fabricated to span a range of lifetimes from sub-nanosecond to over 3.5 ns. These materials were evaluated for FLT, emission intensity, photostability under UV exposure, and fabrication repeatability. The influence of dye concentration and microstructural uniformity was assessed using a confocal microscope. The standards were also applied to validate a chip-on-tip FLIm micro-camera designed for endoscopic imaging.

**Results:**

The dyed epoxy standards demonstrated consistent and reproducible lifetimes, good photostability, and scalable fabrication. Confocal imaging revealed some microstructural heterogeneity, whereas bulk measurements remained robust. The standards enabled effective validation of the FLIm micro-camera, including spatial and temporal resolution assessment, and highlighted platform-dependent biases in lifetime estimation.

**Conclusions:**

Dyed epoxy materials show strong potential as practical, scalable tools for FLIm system calibration and quality assurance. These standards may support cross-platform validation and benchmarking of emerging FLIm technologies and could contribute to the development of future regulatory frameworks for clinical adoption.

## 1 Introduction

Fluorescence lifetime imaging (FLIm) has potential as a powerful diagnostic tool due to its ability to provide contrast based on the intrinsic decay time of fluorophores, independent of the fluorophore concentration or the excitation intensity used.^1^^–^^4^ Unlike fluorescence intensity measurements, which can be influenced by photobleaching, scattering, or absorption, fluorescence lifetime (FLT) measurements can offer a higher biochemical specificity. This makes FLIm particularly valuable in biomedical applications, where subtle changes in tissue composition or metabolic state can be detected through lifetime variations.^2^^,^^4^^,^^5^

Recent studies have demonstrated the clinical potential of FLIm for label-free intraoperative assessment of tumor margins in various tissues, including the brain,^6^^,^^7^ oral cavity,^8^^,^^9^ and breast.^10^^,^^11^ Beyond intraoperative oncology, FLIm has also been explored for dermatological diagnostics,^12^ and for probing subcellular lipid heterogeneity in metabolic studies.^13^ These applications highlight the growing interest in translating FLIm from research settings to clinical environments. This is reflected in the large number of novel systems for *in vivo* FLIm, which have been reported in recent years.^14^ In addition to stand-alone FLIm systems, multimodal approaches have been explored to combine FLIm with complementary techniques such as Raman^15^^–^^17^ and optical coherence tomography^18^^,^^19^ for enhanced tissue characterization.

The development of diagnostic imaging systems more generally has led to the use of tissue-simulating objects, commonly known as “phantoms.”^20^^,^^21^ These phantoms serve multiple critical purposes, including initial system design testing, optimizing signal-to-noise ratios in existing systems, performing routine quality control, and comparing performance between different systems. Phantoms can be fabricated from solid or liquid materials that mimic a range of tissue optical properties as well as anthropomorphic features, enabling realistic evaluation of various system performance.^22^^,^^23^ For established systems in routine clinical use with regulatory approval, bodies such as the American College of Radiology, the American Association of Physicists in Medicine, and the Canadian Organization of Medical Physicists typically recommend or require specific phantoms for validating system performance and ensuring uniformity across institutions and over time.^20^

Given the rapid rate at which new FLIm systems are being developed and the vast amounts of tissue lifetime data being acquired both *in vivo* and *ex vivo*, there is an increased need for standardization and cross-platform validation. A major barrier to clinical translation remains the lack of robust, reproducible standards for system calibration and validation.^24^^,^^25^ Currently, system validation often relies on liquid dye solutions with known lifetimes, such as Rhodamine 6G or Erythrosine B, measured under controlled conditions.^26^^,^^27^ Although effective in laboratory settings, this approach is impractical for routine or on-site calibration, particularly in clinical workflows, and is sensitive to environmental factors such as oxygen quenching and dye aggregation. Biological samples such as Convallaria are often used for qualitative fluorescence lifetime imaging microscopy (FLIM) demonstrations; however, their autofluorescence properties can vary between specimens and degrade over time, making them unsuitable as reproducible calibration standards. Commercial fluorescent plastic slides, available from suppliers such as Thorlabs, are designed to provide broad absorption and emission coverage for alignment and qualitative checks. However, they are not developed for use as lifetime standards. There are FLIM standards available from BioPixS,^28^ but the specific dyes used are not disclosed. As a result, these materials are ill-suited for rigorous calibration or cross-platform validation.

To address these limitations, several studies have focused on improving the accuracy and reproducibility of FLIm calibration; Talbot et al.^29^ identified systematic errors in FLT fitting using the delta function convolution method, particularly when the reference dye has a longer lifetime than the sample, and proposed a correction model to mitigate these biases. Complementing this, Margineanu^25^ provided a comprehensive experimental framework for calibrating time-domain FLIm systems, emphasizing the importance of acquiring accurate instrument response functions (IRF) and using well-characterized reference dyes. Both studies also noted that discrepancies in lifetime measurements across platforms can arise due to differences in photon collection geometry and reabsorption effects, further underscoring the importance of physical standards that can reveal and help correct such biases. Building on this, Freymüller et al.^30^ developed a microstructured phantom incorporating fluorescent beads embedded at known depths within a scattering medium. This design enables depth-resolved calibration and is particularly suited for characterizing axial resolution in multiphoton and microendoscopic systems lacking z-stage control. However, the phantom’s short shelf-life and complex fabrication process limit its scalability for routine use or widespread deployment.

In this work, we present a complementary approach by developing and characterizing a set of solid-state FLT standards using dyed epoxy resins. These standards are designed to be photostable, reproducible, and easy to handle, making them suitable for both research and clinical environments. We fabricate and characterize a range of such materials incorporating different dyes, targeting a spread of lifetimes relevant to biomedical FLIm. The materials are evaluated in terms of their FLT, intensity, photostability, and fabrication repeatability. We also explore the effect of dye quantity and assess the microstructure of the materials using confocal FLIM. Finally, we demonstrate the utility of these standards by applying them to validate a novel chip-on-tip FLIm micro-camera system developed for endoscopic applications.^31^

## 2 Materials and Methods

### 2.1 FLT Standards Fabrication Process

The solid-state FLT standards were fabricated using Poly-optic^®^ 1490 Clear Casting Resin, a water-clear urethane resin consisting of a base (part A) and a curer (part B). The two-part resin was mixed by weight at a 100A:90B ratio, with varying masses of fluorescent dyes added to achieve a range of target lifetimes. In this study, four Smooth-On Ignite™ dyes (Smooth-On, Inc., Macungie, Pennsylvania, United States) have been used: purple (PMS purple), yellow (PMS 809C), magenta (PMS 813C), and orange (PMS 805C). The Smooth-On Ignite™ dyes were selected for their strong visible fluorescence and lifetimes spanning the sub-nanosecond to multinanosecond range. Other dyes from the same range were screened, and four were chosen to cover a clinically relevant lifetime span. The Poly-optic^®^ 1490 resin was selected for its optical clarity and stable curing properties. The wide commercial availability of these components also facilitates reproducible standard fabrication internationally. Solid standards containing each dye were fabricated. The dye quantity added for each standard material is as follows: 119 mg for the purple standards, 219 mg for yellow, 90 mg for magenta, and 160 mg for the orange standards. An epoxy sheet containing no dye was also fabricated and measured to assess the fluorescence of the undyed epoxy and its potential influence on the standard material performance.

**Fig. 1:**
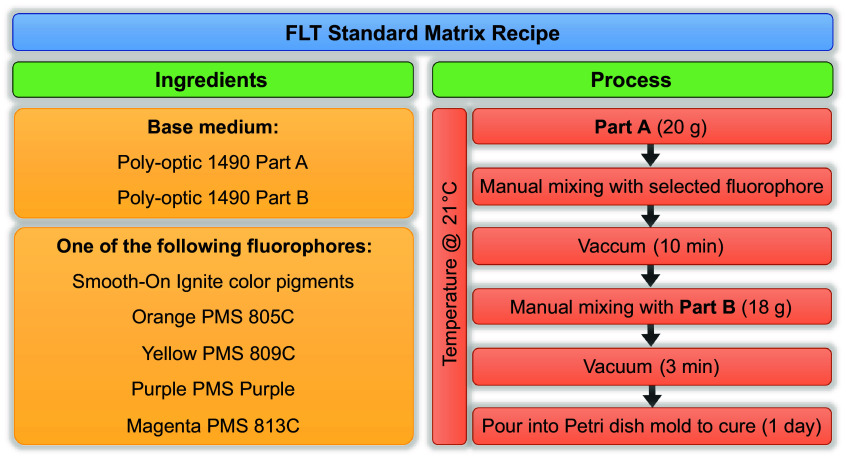
Overview of the solid fluorescence lifetime standard recipe, including all ingredients and the fabrication process steps.

**Fig. 2:**
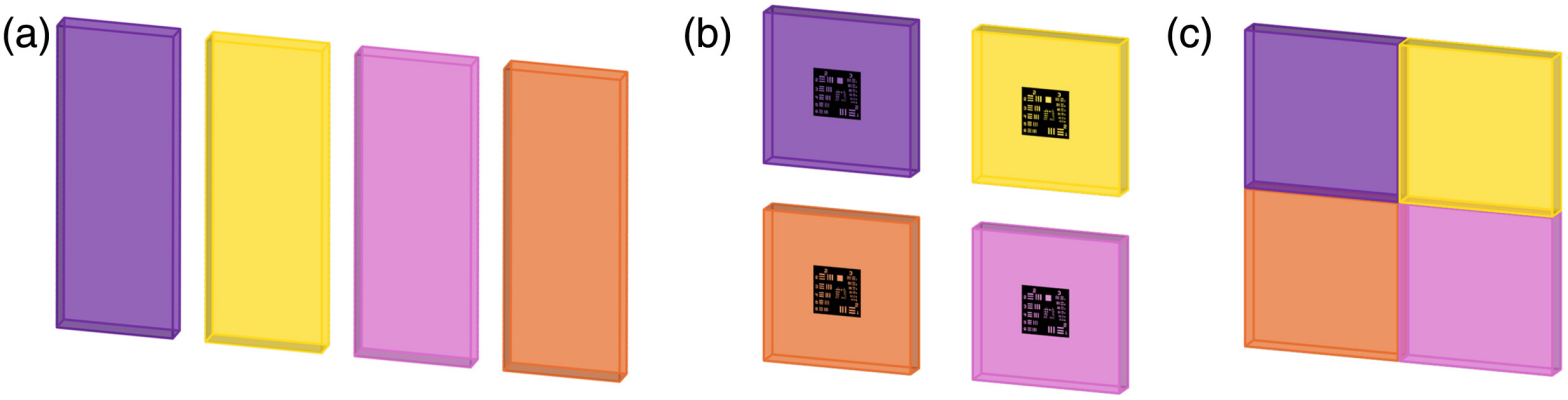
Solid fluorescence lifetime standard tools created (not drawn to scale); 4 standard materials have been tested with a range of lifetimes. The standard sheets can be cut to a desired planar geometry. For this work, use of the standard materials in three forms was explored; (a) as a homogeneous slide cut to 17  mm×45  mm, (b) a square slide cut to 22  mm×22  mm with a chrome USAF resolution test target glued to the surface for combined system evaluation of fluorescence lifetime measurements and spatial resolution, or (c) each material fixed together in a quadrant pattern for system lifetime imaging demonstration with multiple lifetimes in the same field of view.

The fabrication process is illustrated in Fig. 1. First, the selected dye was weighed and thoroughly mixed with part A to ensure uniform dispersion and degassed under vacuum to eliminate air bubbles, which could otherwise scatter light and distort fluorescence measurements. Part B was then added, and the mixture was stirred again to achieve homogeneity. The resulting solution was degassed again. Finally, the mixture was poured into moulds and left to cure undisturbed at room temperature for 16 to 24 h. This process yielded optically clear solid sheets with reproducible fluorescence characteristics. The dyes were selected to span a range of lifetimes, enabling evaluation of FLIm system accuracy across the range of lifetimes covered. The standard sheets can then be cut to a desired planar geometry. For this work, three types of standard tools were explored as illustrated in Fig. 2; (i) a homogeneous slide (ii) a square slide with a chrome USAF resolution test target glued to the surface for combined system evaluation of FLT measurements and spatial resolution (herein referred to as FLT-USAF targets), and (iii) one of each material fixed together for lifetime imaging demonstrations with multiple lifetimes in the same field of view.

### 2.2 Material Characterization

#### 2.2.1 Absorption and emission spectra

The absorption and emission spectra of the dyed epoxy standards were measured to characterize their spectral properties. Spectra were acquired using a fluorescence spectrometer (Cary Eclipse, Agilent, Santa Clara, California, United States) with excitation and emission wavelengths scanned across the visible range.

#### 2.2.2 Fluorescence lifetime system

A home-built FLT system was used for the characterization of the standard materials. This system employs a TCSPC-based single-photon counting architecture similar to that of our well-validated time-domain diffuse optical spectroscopy system.^32^^,^^33^ Measurements were performed using a 405 nm picosecond laser diode (BDL-405-SMN-F, Becker&Hickl, Berlin, Germany) as an excitation source pulsed at a repetition rate of 20 MHz. The materials were excited with a collimated laser beam; fluorescence emission was collected perpendicular to the excitation beam and filtered using a long pass filter with a cut-off wavelength of 500 nm (FELH0500, Thorlabs, Newton, New Jersey, United States). The laser power was adjusted using a variable reflective neutral density filter (NDM4/M, Thorlabs) positioned in the laser beam path before the sample and mounted at an angle to reduce back-reflections. Fluorescence decay curves were recorded using a hybrid photomultiplier detector (PMA Hybrid 50, PicoQuant, Berlin, Germany) and a time-to-digital converter (TDC, Picoharp 300, PicoQuant). A photodiode connected to a power meter (S120VC and PM100D, Thorlabs, Germany) was placed after the sample in the excitation beam path for monitoring of the excitation laser power used for each sample.

For FLT measurements, the laser power was adjusted until a count rate of $10^{5}$ counts per second (cps) ±5% was seen at the hybrid detector. Photon counts were then acquired for one second. The sample was then removed from the excitation beam path, and the laser power was recorded from the power meter.

The system IRF was recorded by removing the long pass filter from the detection path and measuring the laser pulse scattered by a frosted glass microscope slide. The FLT of each sample was found by fitting a monoexponential decay model convolved with the system IRF. Fitting was performed from 80% of the max counts on the rising edge of the measured decay curve to 1% on the falling tail. The code used for data acquisition and fitting is available on GitHub.^34^ The relative fluorescence intensity of each sample was estimated from the inverse of the excitation laser power required to achieve a count rate of $1\times 10^{5}\;\mathrm{cps}$. This is a proxy measurement rather than a direct one, but it gives a useful indication of the relative brightness of the fluorescent materials.

#### 2.2.3 Bulk FLT characterisation

For each sheet of standard material fabricated, three homogeneous slides of 17  mm×45  mm were cut. The three slides were then measured three times each with the system described above, and the mean, standard deviation, and coefficient of variation of these measurements were calculated. The FLT and intensity of the dyes were then compared to assess their suitability for use as solid lifetime standards.

#### 2.2.4 Repeatability

The repeatability of the solid standard fabrication process was assessed by carrying out three fabrication repetitions of the orange standards with 160 mg of orange dye and evaluating the variability in the standard lifetimes across the different repetitions. Three homogenous slides were cut from each sheet, each slide was then measured three times, and the results from each fabrication were compared.

#### 2.2.5 Microscopic inspection

The standard materials were inspected using a Leica TCS SP8 STED3X confocal and super-resolution microscope (Leica Microsystems, Wetzlar, Germany) equipped with the FALCON (Fast Lifetime CONtrast) module for high-speed FLIM acquisition^35^ and processed using LAS X software (Leica Microsystems) at the Advanced Optical Microscopy Facility in the University Health Network, Canada. Samples were excited at 488 nm using a 40 MHz pulsed white light laser with fluorescence emission from 500 to 785 nm detected with a Leica HyD GaAsP single molecule detector. Lifetime images of four homogenous slides, one for each dye, were acquired using a 20× objective for microscopic inspection of the standard materials’ lifetimes and morphologies.

#### 2.2.6 Dye quantity

The influence of the dye quantity on the standard characteristics was also assessed. An additional three orange standards with different quantities of dye were fabricated, resulting in four different quantities of dye added: 80, 160, 320, and 640 mg. As above, three slides were cut from each sheet and measured for comparison. The lifetime and intensity of these samples were then measured with the system described in Sec. 2.2.2. One sample having each dye quantity was also imaged with the confocal system, as described in Sec. 2.2.5, for comparison.

#### 2.2.7 Stability

Photostability is an important factor for the reliability of potential standard materials. To investigate the stability of the materials studied here, the ageing process is simulated through prolonged UV exposure. Four homogeneous slides, one containing each dye, were placed under a high-power 365 nm LED (M365LP1, Thorlabs). The light power density at the samples was measured as $1.35\pm 0.05\;\mathrm{mW}/{\mathrm{cm}}^{2}$. The materials were exposed for a total of 10 h, and the cumulative UV dose was calculated from the measured irradiance and exposure time. Measurements were performed every hour during the 10-h period. At each time point, three lifetime measurements and one intensity measurement were recorded per sample.

### 2.3 Case Study: Characterisation of an FLIm Micro-Camera

As a demonstration of the potential application of the FLT standards, they were used to characterize and validate the wide-field chip-on-tip FLIm micro-camera we have previously reported,^31^ which uses a custom miniaturized 128×120 SPAD array as the image sensor.^36^ The micro-camera sensor has a defective pixel region, which has previously been described^36^; the images presented here have been cropped to remove this defective region. For all micro-camera measurements, photon counts were acquired over 50 time bins, each with a width of 370 ps, and a total acquisition time of ∼30  s. The tail of the measured decay for each pixel was then fit to a mono-exponential model between 90% and 20% of the peak.

FLT images of four uniform standards (one for each dye colour) were captured with the micro-camera. The lifetimes measured for each material by the micro-camera were then compared with the bulk values measured with the system described in Sec. 2.2.2. Lifetime and intensity images of the orange FLT-USAF target were acquired. The intensity image was used to assess the spatial resolution of the system, and the lifetime measured from this target is compared with the orange uniform standard. A lifetime image of the quadrant standard was also captured with the micro-camera to demonstrate the system’s ability to resolve different lifetimes spatially in the same field of view.

## 3 Results and Discussion

### 3.1 FLT Standard Design and Application

**Fig. 3:**
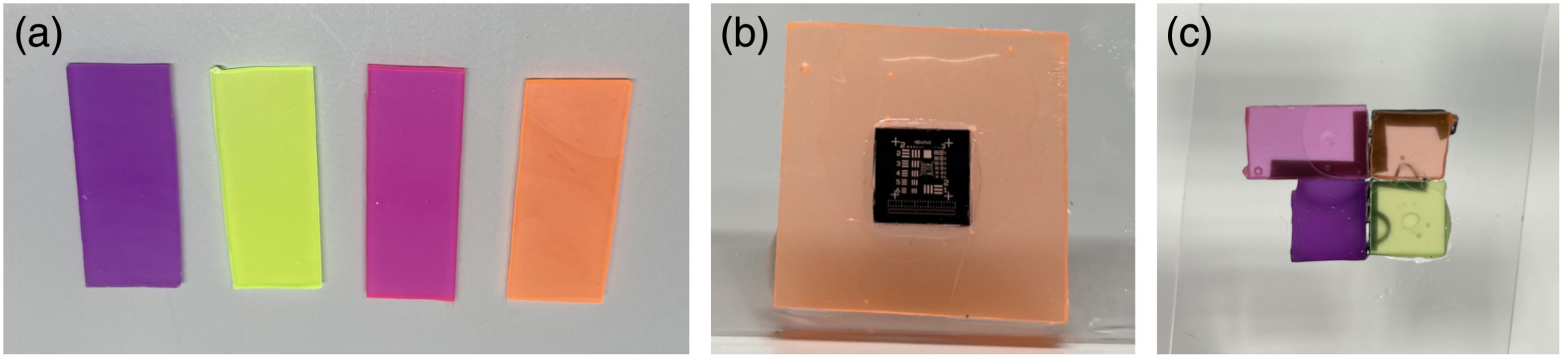
Photographs of the solid-state fluorescence lifetime (FLT) standard tools fabricated using dyed epoxy resins. Three distinct formats were developed to support a range of FLIm system validation tasks: (a) homogeneous slides for bulk lifetime measurements, (b) FLT-USAF targets for simultaneous assessment of spatial resolution and fluorescence lifetime accuracy, and (c) quadrant standards composed of four different dyed materials arranged in a single field of view to enable multi-lifetime imaging demonstrations.

Figure 3 presents photographs of the range of FLT standard tools developed using the dyed epoxy materials. These include homogeneous slides, FLT-USAF targets for combined spatial and temporal resolution assessment, and quadrant standards for multilifetime imaging. These tools enable flexible and application-specific validation of FLIm systems.

### 3.2 Fluorescence Lifetime and Intensity of Standard Materials

**Fig. 4:**
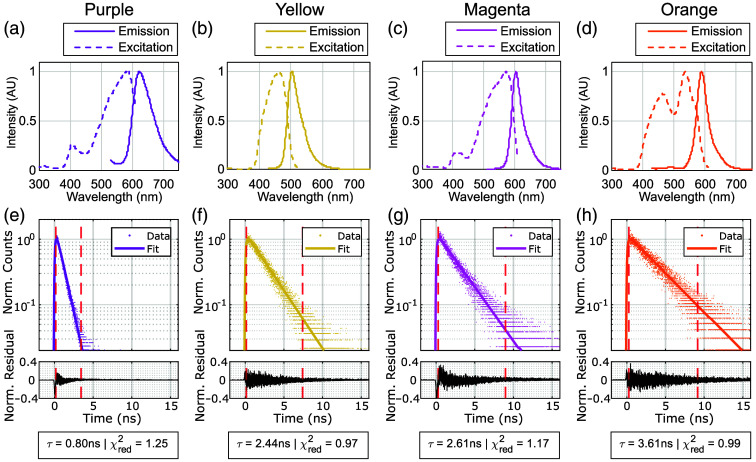
(a)–(d) Normalised absorption and emission spectra of the dyed epoxy standards (purple, yellow, magenta, and orange). (e)–(h) Example plots of the normalized measured decay curves, fit, and normalized residuals for the solid standards fabricated with each dye and measured with the home-built FLT system. The fitted lifetime (τ) and the goodness-of-fit ($\chi_{\text{red}}^{2}$) are also presented for each curve. The dotted red lines in each plot indicate the range of time bins over which the fitting was performed.

Table 1 summarizes the measured FLTs and relative intensities of the four dyed epoxy standards (i.e., purple, yellow, magenta, and orange), along with an undyed control. The absorption and emission spectra for the standards, along with example decay plots and fits are presented in Fig. 4. The results show a clear separation in lifetime values across the different dyes, ranging from 0.80 ns for the purple standard to 3.61 ns for the orange. The undyed epoxy exhibited a low intensity and a lifetime similar to the orange standard, suggesting that the epoxy matrix itself may contribute a weak background signal.

**Table 1:** Measured fluorescence lifetimes and relative intensities of the four dyed epoxy standards (purple, yellow, magenta, and orange), along with an undyed control. The mean, standard deviation (SD), and coefficient of variation (CV) are presented for each.

Dye	Lifetime			Intensity		
	Mean (ns)	SD (ns)	CV (%)	Mean (AU)	SD (AU)	CV (%)
Purple	0.80	0.01	1.25	0.24	0.02	8.33
Yellow	2.43	0.01	0.41	2.48	0.05	2.02
Magenta	2.56	0.05	1.95	0.50	0.01	2.00
Orange	3.61	0.02	0.55	2.29	0.01	0.44
None	3.59	0.12	2.24	0.02	0.003	15.00

However, the background fluorescence from the undyed epoxy was found to be more than an order of magnitude weaker than any of the dyed materials, and approximately 2 orders of magnitude weaker than the yellow and orange standards. This indicates that the contribution of the epoxy matrix to the overall signal is minimal, particularly for the brighter dyes, and should not significantly affect the accuracy of lifetime measurements.

Among the dyed materials, the purple standard had the lowest intensity, but it remains valuable due to its sub-nanosecond lifetime, which is distinct from the other dyes tested. This makes it a useful reference for validating FLIm systems operating in the sub-nanosecond regime, despite its lower brightness. The development of a solid FLT standard with a similarly short lifetime but with enhanced brightness represents a promising direction for future work.

All materials exhibited absorption in the 400 to 500 nm range [as seen in Figs. 4(a)–4(d)], making them suitable for excitation with common sources in this range. The magenta and purple dyes did have lower absorption in this range, consistent with their reduced fluorescence intensity under 405 nm excitation. All dyes except yellow also absorbed in the 500 to 600 nm range, indicating potential for use with longer-wavelength excitation. However, all four of the dyes showed weak fluorescence when excited in the 300 to 400 nm range, which limits their compatibility with FLIm systems using UV excitation (e.g., 355 or 375 nm) targeting endogenous fluorescence from molecules such as NAD(P)H. This spectral gap highlights an area for future development to broaden the standards’ utility in clinical FLIm applications.

### 3.3 Photostability under UV Exposure

**Fig. 5:**
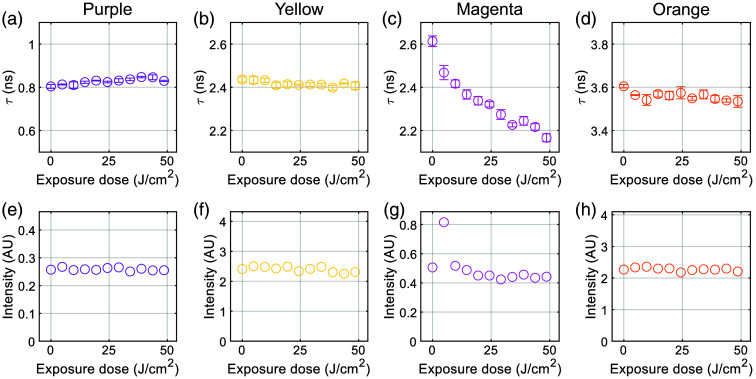
Photostability assessment of the FLT standards under continuous UV illumination over 10 h. Measurements were taken every hour, and the cumulative UV dose is shown on the x-axis. (a)–(d) Fluorescence lifetime of each standard material over the exposure period. Error bars indicate the standard deviation from three measurements at each time point. (e)–(h) Corresponding fluorescence intensity measurements over the same period. The intensity of each standard was only measured once at each time point. The purple, yellow, and orange standards exhibited minimal changes in both lifetime and intensity, indicating good photostability and suitability for long-term use. By contrast, the magenta standard showed a noticeable decline in both parameters, suggesting susceptibility to photodegradation.

The photostability of the standards was evaluated by exposing them to continuous UV illumination over a 10-h period. Measurements were performed at each hour; the corresponding cumulative UV dose was calculated and plotted against the measured lifetime and intensity in Fig. 5; the values of these plots are presented in Table S1 in the Supplementary Material. The FLTs and intensity of the purple, yellow, and orange dyes remained relatively stable throughout the exposure, with minimal drift.

By contrast, the magenta material exhibited a more pronounced change in both FLT and intensity over the exposure period. This suggests that the magenta dye may be more susceptible to photodegradation under prolonged UV illumination. In addition, the magenta standard exhibits a lifetime that is very similar to that of the yellow dye, but with significantly lower fluorescence intensity.

These trends are quantified in Table 2, which summarizes the minimum and maximum values observed for each dye, along with the corresponding percentage changes in both lifetime and intensity. Notably, the magenta intensity measurement after 1 h appears to be an outlier, potentially due to sample positioning or other user-dependent factors during measurement. If this data point is excluded, the percentage variation in magenta intensity is reduced to 22.03%, indicating a less severe degradation than initially observed but still more than twice as large as the other three dyes. Given its reduced photostability, lifetime redundancy with the yellow dye, and weaker signal, the magenta material may be less suitable for continued development as a reference standard.

**Table 2:** Summary of the maximum variation in fluorescence lifetime and intensity observed for each dye during 10 h of continuous UV exposure. The maximum variation represents the difference between the lowest and highest values recorded for each dye over the 10-h exposure period.

Dye	Lifetime			Intensity		
	Min (ns)	Max (ns)	Change (%)	Min (AU)	Max (AU)	Change (%)
Purple	0.80	0.85	5.39	0.25	0.27	6.68
Yellow	2.40	2.44	1.59	2.25	2.50	11.00
Magenta	2.17	2.61	20.65	0.42	0.82	92.50
Orange	3.53	3.60	1.99	2.18	2.36	8.25

These findings have important implications for the practical use of FLT standards in clinical and research environments. The stability of the purple, yellow, and orange dyes under prolonged UV exposure supports their suitability for repeated use in calibration protocols, including routine system checks and longitudinal studies. By contrast, the observed variability in the magenta standard highlights the need for careful dye selection and further optimization to ensure long-term reliability. Overall, photostable standards are essential for maintaining consistency in FLT measurements across time and platforms.

### 3.4 Fabrication Repeatability

To assess the repeatability of the fabrication process, three independent batches of the orange standard were produced using identical protocols. Table 3 shows the mean lifetime and intensity values for each batch. The results demonstrate excellent consistency in lifetime measurements (standard deviation ≤0.03  ns), indicating that the fabrication process is robust and reproducible. Some variation in intensity was observed, which may be attributed to slight differences in dye dispersion or curing conditions.

**Table 3:** Summary of mean lifetime and normalized intensity for the orange standards across three fabrication repetitions. The mean, standard deviation (SD), and coefficient of variation (CV) are presented for each fabrication.

Fabrication no.	Lifetime			Intensity		
	Mean (ns)	SD (ns)	CV (%)	Mean (AU)	SD (AU)	CV (%)
1	3.70	0.03	0.81	3.06	0.06	1.96
2	3.62	0.02	0.55	2.31	0.35	15.15
3	3.61	0.02	0.55	2.29	0.15	6.55

### 3.5 Microscopic Inspection

**Fig. 6:**
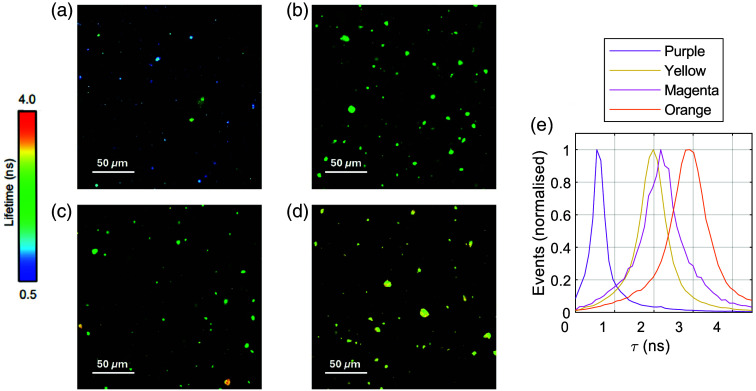
Lifetime images of the (a) purple, (b) yellow, (c) magenta, and (d) orange standards captured with a confocal FLIM microscope using 488 nm excitation, detection between 500 to 785 nm, and a 20× objective. (e) The lifetime histograms obtained from each of these images. The mean lifetimes and standard deviations from these distributions are 0.92±1.25  ns for the purple standard, 2.03±0.80  ns for the yellow standard, 2.32±1.11  ns for the magenta standard, and 2.97±1.11  ns for the orange standard.

Confocal FLIM microscopy was used to inspect the homogeneity of the standards at the microscale. Figure 6 shows lifetime images of each dye captured using a 20× objective. The images reveal a sparse or heterogeneous fluorescence distribution. This may be due to incomplete dissolution of the dye in the epoxy matrix or the presence of insoluble scattering particles in the dye powder. Further optimization of the mixing and filtering process may improve the uniformity of the standards.

The mean lifetimes and standard deviations extracted from the confocal images were 0.92±1.25  ns for the purple standard, 2.03±0.80  ns for the yellow, 2.32±1.11  ns for the magenta, and 2.97±1.11  ns for the orange. Apart from the purple standard, these values are consistently lower than the corresponding bulk lifetimes reported in Table 1.

One factor that may explain the discrepancy is the role of reabsorption and re-emission. In bulk measurements, secondary fluorescence generated by reabsorption of emitted photons and their subsequent re-emission can contribute to the detected signal, effectively extending the measured lifetime. By contrast, the confocal system spatially filters the emission through a pinhole, rejecting fluorescence generated outside the focal volume. As a result, secondary emission occurring away from the confocal spot is unlikely to be detected, and the measured lifetime is not artificially extended. This spatial filtering effect could explain why the confocal measurements yield consistently shorter lifetimes. These findings are consistent with prior reports that highlight platform-dependent biases in FLT estimation due to differences in photon collection geometry and reabsorption effects.^25^^,^^29^

### 3.6 Effect of Dye Quantity

**Fig. 7:**
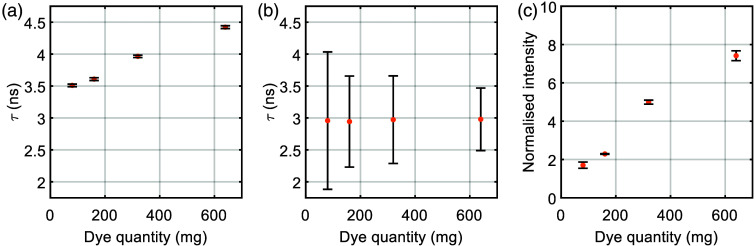
Influence of dye concentration on the fluorescence properties of the orange epoxy standard. Mean fluorescence lifetimes of orange standards with increasing dye concentration measured with the bulk system described in Sec. 2.2.2 (a), and measured with the Leica confocal system (b). (c) Corresponding fluorescence intensities, which increase with dye concentration. Error bars indicate the standard deviation.

The influence of dye quantity on FLT and intensity was investigated using orange standards fabricated with increasing dye quantities. As shown in Fig. 7, the fluorescence intensity increases with dye quantity as expected. However, the bulk TCSPC system measured a pronounced rise in lifetime at higher dye loads, whereas the lifetimes measured with the confocal FLIM system remained consistent. The confocal FLIM images of the four standards and their corresponding lifetime distributions can be seen in Fig. S1 in the Supplementary Material. The data plotted graphically in Fig. 7 is shown in Table S2 in the Supplementary Material. This divergence supports the interpretation that reabsorption and re-emission processes at elevated dye densities contribute to extended apparent lifetimes in bulk measurements, whereas the spatial filtering inherent to confocal imaging suppresses these secondary emissions.

This effect has previously been reported for other fluorophores, such as phthalocyanines,^37^ and these results confirm its relevance for solid-state standards. Although higher dye concentrations can enhance signal strength, they also introduce a concentration-dependent lifetime shift for nonconfocal systems, which should be considered when designing standards for absolute calibration. For future development, it would be valuable to investigate at what fluorophore concentrations the measured lifetimes converge between bulk and confocal systems as this could provide insight into the impact of reabsorption and re-emission effects.

### 3.7 Case Study: FLIm Micro-Camera Validation

**Fig. 8:**
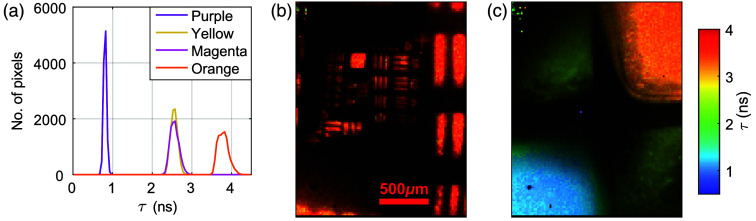
(a) Lifetime histograms from images acquired of each of the uniform standard materials using the microcamera. The mean lifetime measured with the micro-camera was 0.81±0.06  ns for the purple standard, 2.56±0.10  ns for the yellow, 2.57±0.13  ns for the magenta, and 3.80±0.16  ns for the orange. The uncertainty is the standard deviation for each measurement. Lifetime images of (b) the orange FLT-USAF standard and (c) the quadrant lifetime standard captured with the micro-camera, with pixel brightness scaled by the intensity. The mean lifetime obtained from the USAF standard was 3.72±0.26  ns. The quadrant standard is in the same orientation, as shown in Fig. 3(c).

To demonstrate the practical utility of the standards, they were used to validate a wide-field chip-on-tip FLIm micro-camera system. Figure 8 shows lifetime histograms and images acquired using the micro-camera. The micro-camera was clearly able to differentiate the different standard materials based on lifetime with the exception of the yellow and magenta materials, which have been noted to have already been seen to have very similar lifetimes.

The measured lifetimes for each dye were in good agreement with the bulk values obtained from the TCSPC system, although the micro-camera measured slightly longer lifetimes for the yellow and orange standards. This discrepancy is unlikely to be caused by differences in the IRF, as such an effect would impact all dyes similarly. Instead, it may also be attributed to reabsorption and re-emission processes, which are more pronounced for the brightest dyes (yellow and orange). Wide-field imaging systems such as the micro-camera collect fluorescence from a larger volume with no spatial filtering, increasing the likelihood of detecting secondary photons that extend the apparent decay time.

The quadrant reference standard enabled simultaneous imaging of multiple lifetimes in a single field of view, whereas the FLT-USAF target provided a means to assess both spatial resolution and lifetime fidelity. In the previous publication on the micro-camera, the spatial performance was characterized using a standard USAF resolution target and a diffuse white light source from behind; the minimum contrast achieved for group 4, element 2 was 28.4%.^31^ In this work, using the fluorescent resolution target and delivering excitation through the optical fiber, the minimum contrast for the same element was reduced to 12.2%. This reduction in contrast may be attributed to several factors. First, the fluorescence emission is inherently weaker and more spatially diffuse than direct back-illumination with white light, leading to lower signal-to-noise ratios at fine spatial scales. Second, the excitation geometry, delivering light via optical fibre, introduces uneven illumination, which can impact the spatial resolution. These factors combined likely contribute to the reduced contrast observed in the fluorescent imaging configuration. Despite this, the FLT-USAF target still enabled clear resolution of spatial features and provided a valuable tool for assessing the combined spatial and temporal performance of the FLIm system under realistic imaging conditions.

### 3.8 Limitations and Future Prospects

Although the dyed epoxy standards developed here provide robust FLT references, they do not replicate the optical scattering and absorption properties of biological tissues. Such phantoms would allow for more accurate benchmarking of FLIm systems under conditions that mimic *in vivo* imaging. Future work may explore integrating scattering agents and absorbers with the dyed epoxy standards to create hybrid phantoms that combine FLT calibration with tissue-mimicking optical properties.

As mentioned in Sec. 3.2, the current standards exhibited weak fluorescence when excited in the 300 to 400 nm range, which limits their compatibility with FLIm systems employing UV excitation for endogenous fluorophore detection. Future work should explore alternative dyes with enhanced UV absorption to broaden the applicability of the standards.

In addition to hardware validation, the development of standardized analysis protocols for FLIm data is a critical need for ensuring reproducibility and comparability across studies. Recent efforts from Georgakoudi et al.^38^ have sought to establish consensus guidelines for fluorescence-based metabolic imaging, emphasizing the need for standardized acquisition, calibration, and reporting protocols to enhance reproducibility and comparability across systems. Although our work does not directly address this aspect, the reproducible and well-characterized standards presented here could play a supporting role in the development of such protocols by providing a consistent reference for evaluating and calibrating measurement and analysis pipelines.

## 4 Conclusion

This work aimed to address the lack of robust, reproducible standards for FLIm system validation. To do this, a set of solid-state FLT standards has been developed using dyed epoxy resins, targeting a range of lifetimes relevant to biomedical FLIm.

The fabricated standards exhibited lifetimes spanning from sub-nanosecond to over 3.5 ns, with good photostability and reproducibility across fabrication batches. We demonstrated their suitability for system benchmarking through bulk lifetime measurements, photostability testing, and microscopic inspection. Although some heterogeneity in dye distribution was observed at the microscale, the standards provided consistent bulk measurements and proved effective for the validation of a miniaturized chip-on-tip FLIm micro-camera.

In addition to lifetime and intensity characterization, absorption and emission spectra were measured for each material. All dyes showed absorption in the 400 to 500 nm range, with most also absorbing in the 500 to 600 nm range, supporting their compatibility with a range of visible excitation sources. However, the lack of absorption below 400 nm limits their use with FLIm systems employing 355 or 375 nm excitation, which are commonly used for probing endogenous fluorescence. Addressing this spectral limitation will be an important focus for future material development.

These findings support the use of dyed epoxy resins as scalable, application-specific reference standards for FLIm system validation. They may facilitate the development of benchmarking protocols for emerging FLIm-based diagnostic tools and support future regulatory frameworks for clinical FLIm instrumentation. Future work will focus on improving material homogeneity, expanding the range of available lifetimes and excitation compatibility, and developing protocols for system benchmarking in both clinical and research settings.

## Supplementary Material

10.1117/1.JBO.31.11.113503.s01

## Data Availability

All data and code used for analysis in this paper are publicly available on Zenodo (https://doi.org/10.5281/zenodo.17952538).
