# Supplementary material for: Development of solid-state fluorescence lifetime standards for clinical applications using dyed epoxy resins
Source: J Biomed Opt. 2026 Mar 24;31(11):113503. doi: 10.1117/1.JBO.31.11.113503 (PMC13012335; doi:10.1117/1.JBO.31.11.113503)
Supplement: Supplementary file 1 [file JBO_031_113503_SD001.docx]

Supporting Information:

Development of solid-state fluorescence lifetime standards for clinical applications using dyed epoxy resins

Dario Angelonea,*, Keela Hughes^a,b^, Hasti Yavari^a^, Sara Eugenia Garduño Gómez^c^, Brian C. Wilson^d^, Stefan Andersson-Engelsa,b, Katarzyna Komolibus^a^, Sanathana Konugolu Venkata Sekar^a,b,^*^[[1]](#footnote-1)^*

*aBiophotonics, Tyndall National Institute, Lee Maltings Complex, Cork, T12 R5CP, Ireland*

*bSchool of Physics, University College Cork, College Road, Cork, Ireland*

*cBioPixS Ltd. (Biophotonics Standards), Lee Maltings Complex, Cork T12 R5CP, Ireland*

*dPrincess Margaret Cancer Centre, University Health Network, Toronto, ON M5G 1L7, Ontario, Canada*

**Table S1**. Lifetimes ($\boldsymbol{\tau}$) and intensities ($\boldsymbol{I}$) of the dye materials under prolonged UV exposure measured with the TCSPC-based system described in Sec. 2.2.2. These results are plotted in Fig. 5.

| **Time (hr)** | **Dose (J/cm^2^)** | **Purple** | | **Yellow** | | **Magenta** | | **Orange** | |
| --- | --- | --- | --- | --- | --- | --- | --- | --- | --- |
|  |  | $\boldsymbol{\tau}$ **(ns)** | $\mathbf{I}$ **(AU)** | $\boldsymbol{\tau}$ **(ns)** | $\mathbf{I}$ **(AU)** | $\boldsymbol{\tau}$ **(ns)** | $\mathbf{I}$ **(AU)** | $\boldsymbol{\tau}$ **(ns)** | $\mathbf{I}$ **(AU)** |
| 0 | 0.00 | 0.80 ± 0.01 | 0.26 | 2.44 ± 0.01 | 2.41 | 2.61 ± 0.02 | 0.51 | 3.60 ± 0.01 | 2.27 |
| 1 | 4.86 | 0.81 ± 0.00 | 0.27 | 2.43 ± 0.02 | 2.50 | 2.46 ± 0.03 | 0.82 | 3.56 ± 0.00 | 2.34 |
| 2 | 9.72 | 0.81 ± 0.02 | 0.26 | 2.43 ± 0.01 | 2.48 | 2.42 ± 0.02 | 0.52 | 3.54 ± 0.03 | 2.36 |
| 3 | 14.58 | 0.82 ± 0.01 | 0.26 | 2.41 ± 0.01 | 2.43 | 2.37 ± 0.02 | 0.49 | 3.57 ± 0.01 | 2.30 |
| 4 | 19.44 | 0.83 ± 0.00 | 0.26 | 2.41 ± 0.01 | 2.49 | 2.34 ± 0.02 | 0.45 | 3.56 ± 0.02 | 2.30 |
| 5 | 24.30 | 0.82 ± 0.00 | 0.26 | 2.41 ± 0.00 | 2.34 | 2.32 ± 0.01 | 0.45 | 3.57 ± 0.03 | 2.18 |
| 6 | 29.16 | 0.83 ± 0.01 | 0.27 | 2.41 ± 0.01 | 2.42 | 2.27 ± 0.02 | 0.42 | 3.55 ± 0.01 | 2.25 |
| 7 | 34.02 | 0.84 ± 0.01 | 0.25 | 2.41 ± 0.01 | 2.48 | 2.23 ± 0.01 | 0.44 | 3.57 ± 0.02 | 2.28 |
| 8 | 38.88 | 0.85 ± 0.00 | 0.26 | 2.40 ± 0.01 | 2.30 | 2.24 ± 0.02 | 0.46 | 3.55 ± 0.01 | 2.27 |
| 9 | 43.74 | 0.85 ± 0.01 | 0.25 | 2.42 ± 0.00 | 2.25 | 2.21 ± 0.01 | 0.43 | 3.54 ± 0.01 | 2.30 |
| 10 | 48.60 | 0.83 ± 0.00 | 0.26 | 2.41 ± 0.02 | 2.31 | 2.16 ± 0.02 | 0.44 | 3.53 ± 0.03 | 2.21 |

**Table S2** Mean fluorescence lifetimes and intensities of orange standards fabricated with varying dye quantities, along with their standard deviations (SD). Bulk measurements were acquired using the custom TCSPC-based system (Sec. 2.2.2), and confocal lifetimes were obtained with a Leica confocal microscope (Sec. 2.2.5). Corresponding confocal images are shown in Fig. S1. Data are plotted graphically in Fig. 7.

| **Dye Quantity (mg)** | **Bulk Lifetime (ns)** | | **Confocal Lifetime (ns)** | | **Intensity (AU)** | |
| --- | --- | --- | --- | --- | --- | --- |
|  | **Mean** | **SD** | **Mean** | **SD** | **Mean** | **SD** |
| 80 | 3.51 | 0.02 | 2.96 | 1.08 | 1.70 | 0.16 |
| 160 | 3.61 | 0.02 | 2.94 | 0.71 | 2.29 | 0.02 |
| 320 | 3.96 | 0.02 | 2.97 | 0.69 | 4.99 | 0.10 |
| 640 | 4.42 | 0.02 | 2.98 | 0.49 | 7.41 | 0.26 |


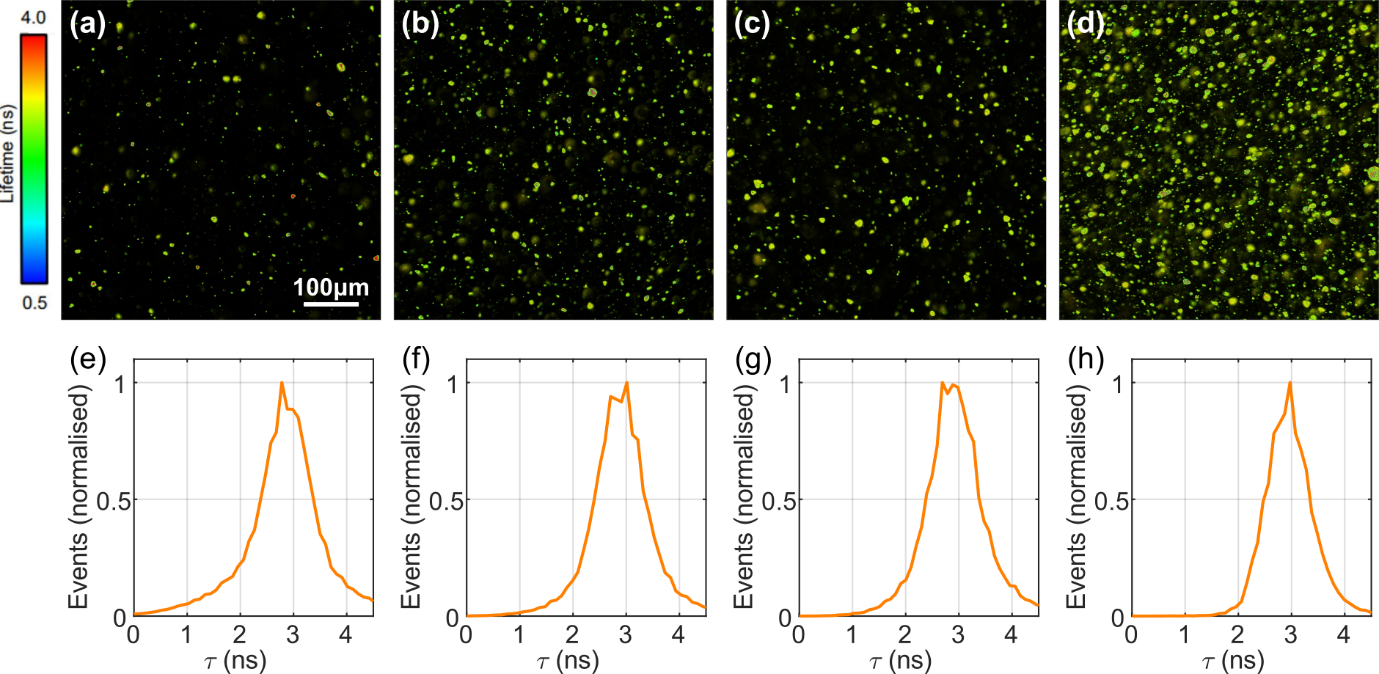


**Figure S1.** Lifetime images of orange standards with (a) 80 mg, (b) 160 mg, (c) 320 mg, and (d) 640 mg. Images captured with a confocal FLIM system using 488 nm excitation, detection between 500 nm and 785 nm. Corresponding lifetime distributions.

1. Corresponding authors, *email:* [dario.angelone@tyndall.ie](mailto:dario.angelone@tyndall.ie), [sanathana.konugolu@tyndall.ie](mailto:sanathana.konugolu@tyndall.ie) [↑](#footnote-ref-1)
